# *Madurella mycetomatis* infection of the foot: a case report of a neglected tropical disease in a non-endemic region

**DOI:** 10.1186/s12895-019-0097-1

**Published:** 2020-01-10

**Authors:** Basma Karrakchou, Ibtissam Boubnane, Karima Senouci, Badreddine Hassam

**Affiliations:** Dermatology and Venereology Department, Ibn Sina Hospital, Mohammed V University, Rabat, Morocco

**Keywords:** *Madurella mycetomatis*, Mycetoma, Actinomycetoma, Eumycetoma, Neglected disease

## Abstract

**Background:**

Mycetoma is an uncommon chronic granulomatous infection of cutaneous and subcutaneous tissues that can be caused by filamentous bacteria (actinomycetoma) or fungi (eumycetoma). It is the prerogative of young men between the third and fourth decade and is transmitted through any trauma causing an inoculating point. The classic clinical triad associates a painless hard and swelling subcutaneous mass, multiple fistulas, and the pathognomonic discharge of grains. Although endemic in many tropical and subtropical countries, mycetoma can also be found in non-endemic areas as in Morocco, and causes then diagnosis problems leading to long lasting complications. Therefore, we should raise awareness of this neglected disease for an earlier management. Under medical treatment however, mycetoma has a slow healing and surgery is often needed, and relapses are possible.

**Case presentation:**

Herein we report a case of a 64 years old patient, with a history of eumycetoma occurring ten years ago treated with oral terbinafine coupled with surgery. A complete remission was seen after 2 years. He presented a relapse on the previous scar 6 months ago. There wasn’t any bone involvement in the magnetic resonance imaging (MRI). The patient was put under oral terbinafine with a slow but positive outcome.

**Conclusion:**

Through this case report, we perform a literature review and highlight the importance of increase awareness of mycetoma in clinical practice especially in non-endemic regions.

## Background

Mycetoma is a chronic granulomatous infection of cutaneous and subcutaneous tissues that can be caused by filamentous bacteria (actinomycetoma) or fungi (eumycetoma) [1]. It occurs typically in young men between 20 and 40 years old, and is the prerogative of farmers who are exposed to contaminated soil during minor injuries in most cases [2]. Clinically, mycetoma or « Madura foot » is an inflammatory tumor, often polyfistulised, evolving in a chronic mode. Its fistulas give rise to grains whose color directs towards the causative germ [3, 4]. Although the true incidence of mycetoma remains uncertain, 60% of mycetoma are bacterial and 40% are fungal [5]. And most of the cases fall between latitude 15°S and 30°N, the so-called “mycetoma belt” characterized by warm, dry, semi-desert regions with low rainfall [3, 6]. Therefore, mycetoma is endemic in many countries in the tropics and subtropics [3], but little known in other countries including Morocco where they prevail in sporadic forms and cause diagnostic problems [7].

In this work, a case of eumycetoma of the foot in a Moroccan patient was reported, and literature review was provided.

### Case presentation

Our patient is a 64 years old man from Sidi Slimane in the center of Morocco where he currently lives and works as a farmer. He is married and has five children in good health. He presented ten years ago a history of a nodule located in the dorsal surface of his right forefoot, which has progressively increased in size until becoming a swelling and slightly painful polyfistulised tumor emitting dark grains. No previous trauma or injury of the foot has been noticed by the patient and no travel to endemic zone in Africa or outside Africa has been reported. He was hospitalized in the Dermatology department of the University Hospital Ibn Sina of Rabat, and the diagnosis of eumycetoma due to *Madurella mycetomatis* was detected. The patient was initially treated with oral itraconazole at a dosage of 400 mg/day during 2 years. After antifungal treatment, the lesions did not improve substantially and itraconazole was substituted by terbinafine 500 mg/day, associated with surgical debridement of the tumor. The outcome was good with total healing of the lesions, and treatment with terbinafine was continued to achieve a total length of 6 months.

**Fig. 1:**
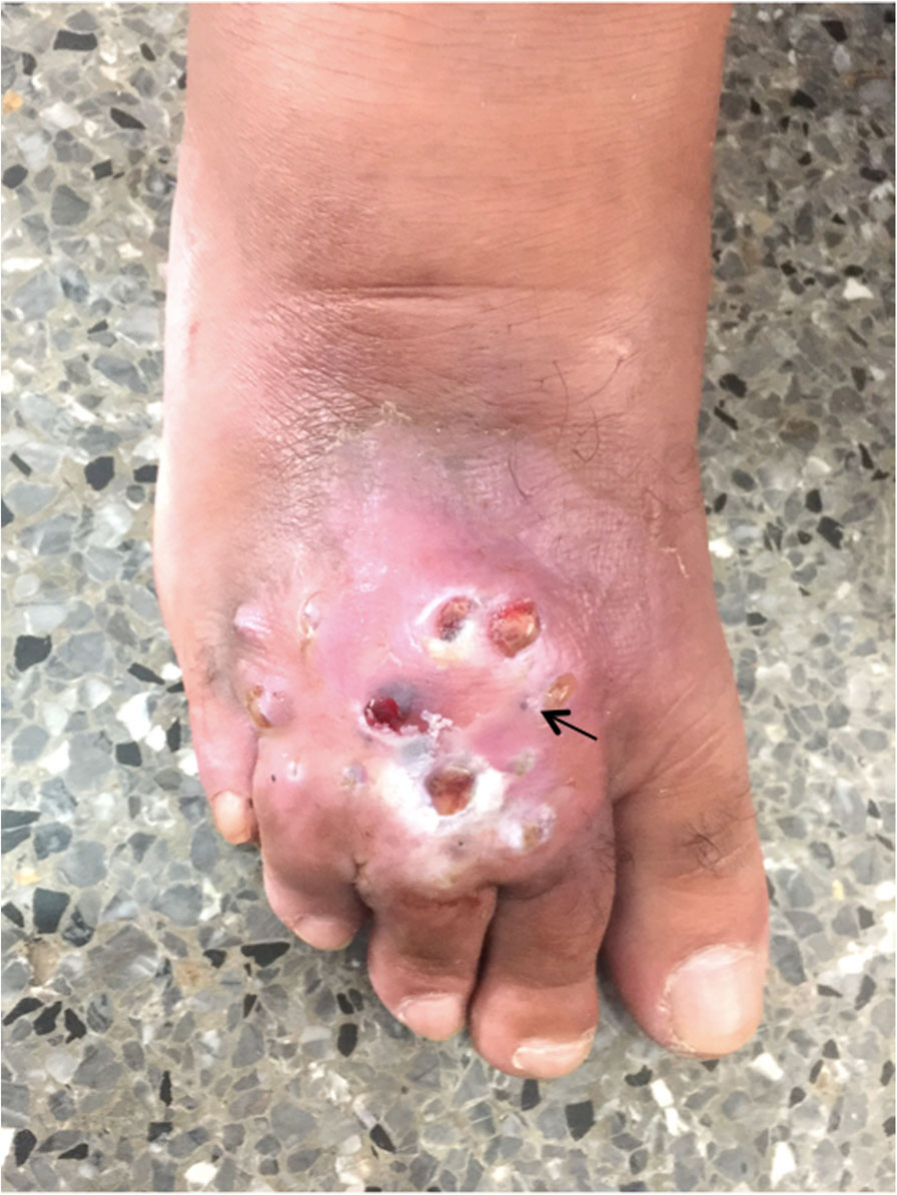
Mycetoma of the right forefoot with polyfistulas emitting serohematic fluid and dark grains (arrow)

The current history goes back to 6 months by the appearance of a nodule localized on the previous scar, having the same evolution than the previous one. On physical examination, he had an indurated inflammatory tumor of the dorsal surface of the right forefoot, measuring 15x10cm, adherent to the skin and to deep structures, with many visible openings, which let emerge seropurulent sometimes hematic fluid and small black grains of 1 to 2 mm (Fig. 1). Moreover, inflammatory inguinal lymphadenopathy was found in the right side, and macerated toes intertrigos were present in the right foot. Furthermore, there was no fever or alteration of the general condition.

**Fig. 2:**
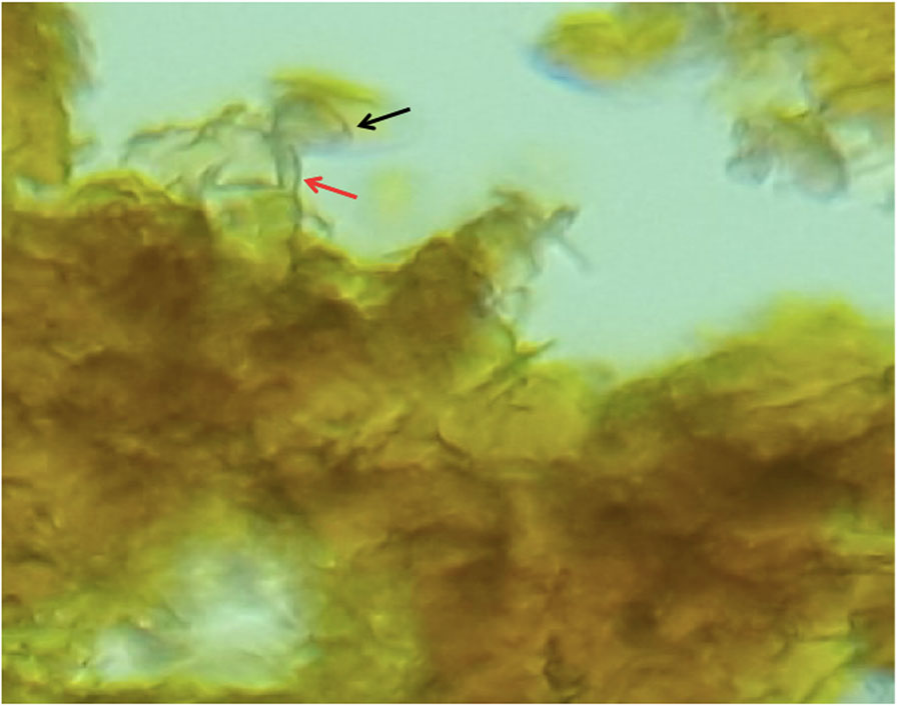
Direct examination of black grains under optical microscopy × 40 (Parasitology and Mycology Department, Ibn Sina Hospital, Rabat). Evocative aspect of *Madurella mycetomatis*: thick septated and branched hyphae (red arrow), ending in vesicles corresponding to circular chlamydoconidia (black arrow)

**Fig. 3:**
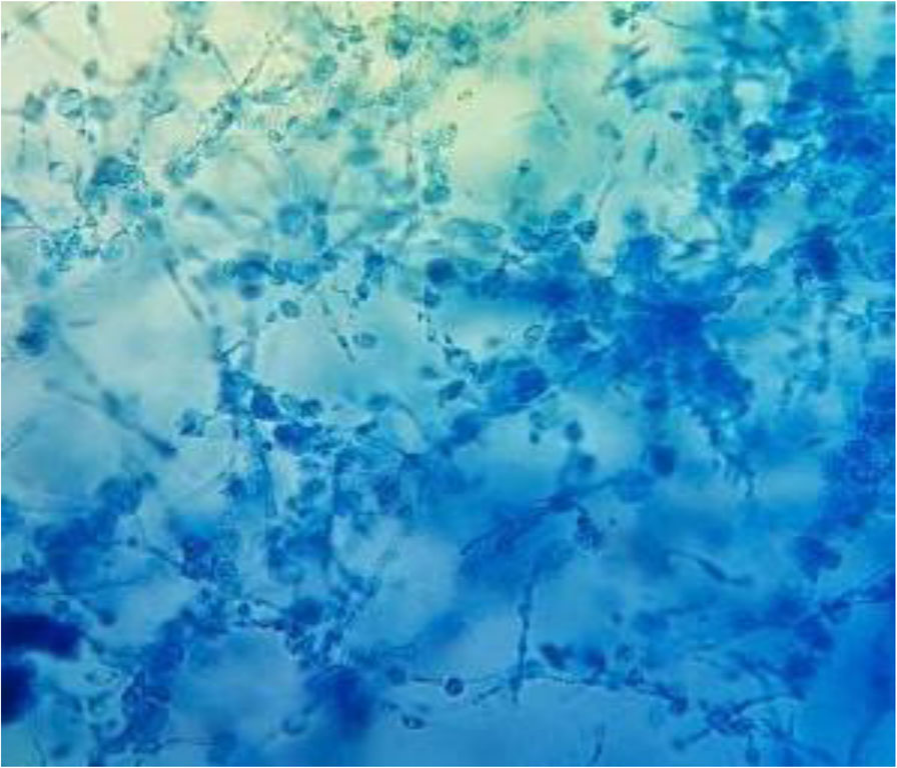
Microscopic aspect of *Madurella mycetomatis’* colony colored with Lactophenol Cotton Blue Stain × 40 (Parasitology and Mycology Department, Ibn Sina Hospital, Rabat)

**Fig. 4:**
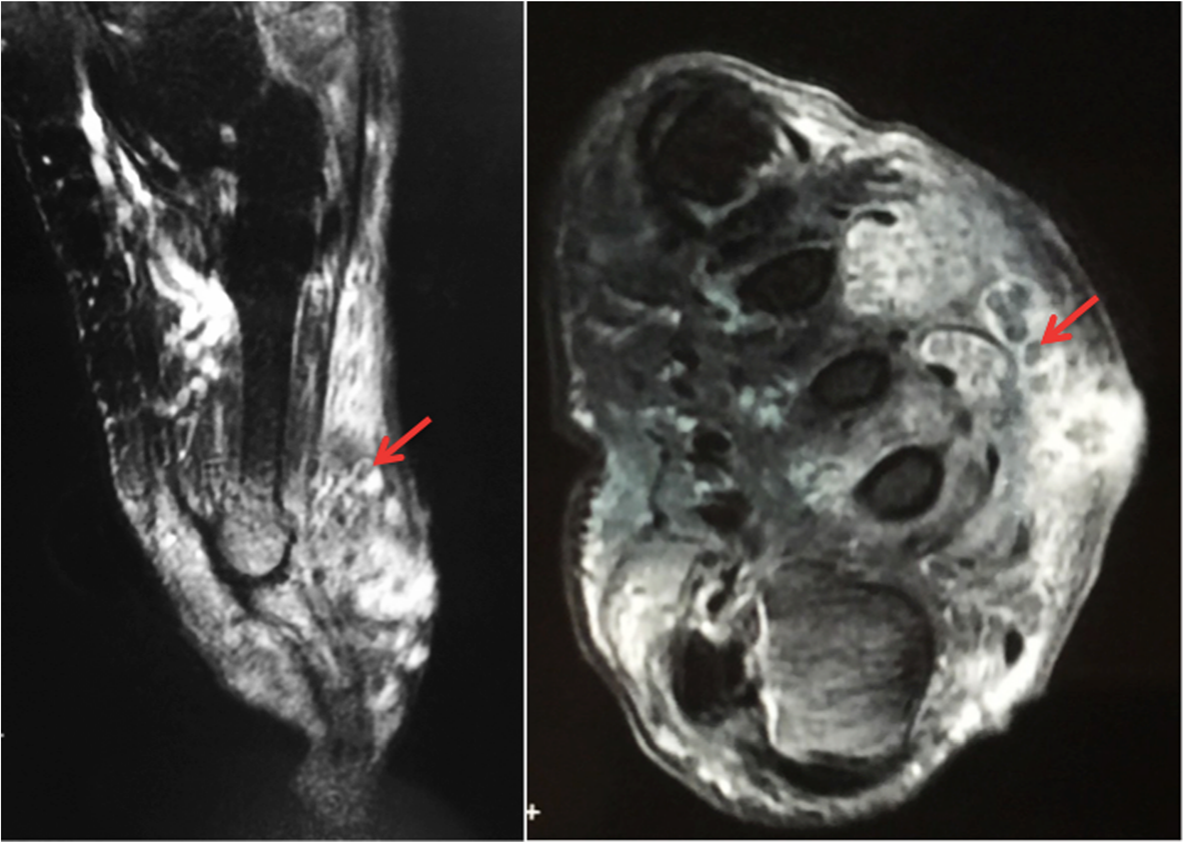
Magnetic resonance imaging scan of the right foot showing the « dot in circle » sign (arrow). a-Sagittal view; b-Transversal view

On the other hand, several differential diagnoses of mycetoma such as cutaneous tuberculosis, profound mycosis, fistulised osteitis, leprosy, and cutaneous metastatic lesions were considered. Complementary examinations showed a normal aspect on foot X-rays. The biological assessment didn’t show any signs of bacterial infection. A biopsy was performed and during this procedure, a serohematic discharge was witnessed with conglomerates of small and firm blackish pellets, evoking eumycetoma (Fig. 1). Tissue and black grain samples were analyzed for bacterial, mycological and histological evaluation. The anatomopathological aspect showed a polymorphic inflammatory cell reaction. The bacterial analysis was negative, and the mycological study revealed on direct examination septated hyphae containing numerous chlamydoconidia measuring 2 to 5 μm in diameter with terminal dilatations giving a vesicle appearance (Fig. 2). The fungal culture on Sabouraud media established the diagnosis of certainty and identified *Madurella mycetomatis* after 3 weeks of growth (Fig. 3). A magnetic resonance imaging of the right foot was performed to determine the lesions extension. This examination identified multiple collections of the right forefoot soft tissues, fusing along extensor and flexor tendons without associated joint or bone invasion (Fig. 4). A treatment with oral terbinafine at a dosage of 750 mg/day for at least 1 year was started because of its previous efficiency in our patient. We performed monthly liver function tests (liver transaminases) to assess treatment tolerability. Within 6 months, the lesions evolution was slow with a fistulas drying up, and no liver damage was noted.

### Discussion and conclusions

Mycetoma was first reported in 1843 by Dr. John Gill in Madurai, India [8] and was then called “Madura foot”. It is a chronic granulomatous infection of cutaneous and subcutaneous tissues that can be caused by filamentous bacteria (actinomycetoma) or fungi (eumycetoma) [1]. Indeed, a bacterial origin is found in 60%, whereas fungi are responsible of 40% of cases reported worldwide [2]. Moreover, causative bacterial agents are more numerous than fungal agents (only four agents reported) [9] (Table 1). The mycetoma’s agents are found throughout the world, while they are endemic in the tropical and subtropical zones with hot and dry climates, and intermittent short periods of rainfall. These zones are called “mycetoma belt” and they include Mauritania, Senegal, Chad, Sudan, Ethiopia, Somalia, Yemen, India, Thailand, Mexico, and the Bolivarian Republic of Venezuela. Therefore the World Health Organization approved (May 2016) a resolution (WHA69.21) recognizing mycetoma as a neglected tropical disease because of its endemicity in poor populations living in remote areas of developing countries [1]. However, some sporadic autochthone cases have been described in temperate zones such as in the Maghreb, where the disease incidence is rare, as in Morocco, causing diagnosis problems.

**Table 1:** Clinical, radiological and microbiological differential features between eumycetoma and actinomycetoma

	Eumycetoma	Actinomycetoma
Causative organism	=Fungi, mainly four *Madurella mycetomatis* (most common) *Madurella grisea* *Pseudoallescheria boydii* *Leptosphaeria senegalensis* Others …	=Bacteria, numerous *Nocardia spp*. (mostly in regions with higher humidity) *Nocardia brasiliensis* *Nocardia asteroides* *Nocardia otidiscaviarum* *Actinomadura spp* *Actinomadura madurae* *Actinomadura pelletieri* *Streptomyces somaliensis* *Actinomyces israeli* Others …
Clinical	Slow evolution Most often the foot Well-limited tumor with a clear margin White or black grains Rare lymphatic metastasis	Rapid evolution Most commonly the foot, but also the chest, head and abdomen Diffuse mass with no clear margin, more inflammatory and destructive Many grain colors, but not black Frequent lymphatic metastasis
MRI	Few but large well limited soft tissues cavities Less osteophilic	Numerous soft tissues cavities with a small size and unclear margins Rapid bone involvement
Direct microscopic examination of the grains	Black or white Diameter > 3 μm Few thick hyphae dilated in places to form vesicles No fringes Periodic-acid–Schiff stain Gomori methanamine silver stain	Red or white Diameter < 1 μm Thin and numerous grains, no hyphae Fringes in the outskirt Gram stain
Culture of the grains	Sabouraud - antibiotics culture media Slow growth 2–3 weeks	Sabourauld without antibiotics media Loewenstein culture media Rapid growth

Mycetoma is the prerogative of young adults, mostly men between the third and fourth decade with a sex ratio of 4/1 [5]. The transmission is done via a contact with a contaminated soil through a thorn prick or any trauma causing an inoculating point [1]. However, the infection is not transmitted from one person to another. Therefore, mycetoma is found generally in rural areas and affects manual workers or those who walk barefoot, such as farmers, laborers, and herdsmen. Furthermore, our patient corresponds to the description of the persons at risk to develop the disease in view of his sex, origin, and work.

The classic clinical triad associating a painless hard and swelling subcutaneous mass, multiple fistulas, and discharge of grains characterizes mycetoma. The foot is the most common site of involvement, as seen in our patient and as indicated by the denomination “Madura foot” [10]. Nonetheless, all other parts of the body can be affected such as the arm, forearm, hand, back, thorax, head and neck [11]. The physiopathological steps are characterized by four phases. A first long period of incubation characterizes this disease, going from a few weeks to several years (6 months in our patient). Then appears a discomfort and pain, followed by the constitution of a slightly inflammatory subcutaneous nodule of 1 to 4 cm of diameter, gradually increasing in size. At the state phase, the infection extends to superficial structures, and fistulises to the skin, leaving grains whose color directs toward the causative germ (Table 2). The classic Madura tumor is then constituted and usually is superinfected leading sometimes to fatal sepsis. Left untreated, the infection spreads through deep structures including fascia planes and the underlying bone and muscle. Lymphatic extension has been reported in few cases, as in our patient who presented with right inguinal lymphadenopathy [12]. It is to highlight that the clinical aspects of eumycetoma and actinomycetoma are almost similar, with few differences (Table 1).

**Table 2:** Main mycetoma agents depending on the color of the grains

Dark grains =fungi mycetoma	*Madurella mycetomatis* (Sahelian Africa, Middle East, India) *Madurella grisea* (South America) *Leptosphaeria senegalensis* (Mauritania, Senegal) *Leptosphaeria tompkinsii* (Exceptional) *Pyrenochaeta reomeroi* (Rare) *Exophiala jeanselni* (Rare)
Red grains =bacterial mycetoma	*Actinomadura pelletieri* (West Africa)
White and yellow grains	*Actinomadura madurae* (Cosmopolitan) *Streptomyces somalienis* (Desert and subdesertic regions) *Nocardia spp* (Tropical humid regions)
White grains	*Pseudallescheria budii* (Quite rare, tropical and temperate regions) *Acremonium spp* (Rare) *Fusarium spp* (Rare) *Neotestudina rosatii* (Exceptional) *Aspergillus spp* (Exceptional) *Dermatophytes* (Rare)

Differential diagnoses of mycetoma mainly include other subcutaneous infections with similar presentations. Cutaneous tuberculosis, especially gumma of the foot, represents the main differential diagnosis in our context, as Morocco remains an endemic country where tuberculosis still causes ravages. Other atypical mycobacteriosis, blastomycosis, soft tissue tumors, and chronic fistulised osteomyelitis can have the same clinical presentation but the pathognomonic presence of grains rectifies the diagnosis [13]. Recently, dermoscopy has proven its usefulness in detecting subclinical grains. It gives a clinical diagnosis presumption by showing structureless blue-white areas in eumycetoma, corresponding to deep black grain localization. A white halo surrounds these areas, and sometimes polymorphic vessels are seen [14].

The mycetoma diagnosis is primarily clinical. Additional tests are performed, on the one hand, to determine the causative organism for appropriate treatment, and on the other hand to look for the tumor extension. Direct microscopic examination of the grains can already give an etiological orientation. The grains are collected with a scalpel and placed between blade and lamella with the KOH solution. The size, color, consistency of grains, and the existence of cement makes it possible to distinguish fungal grains from actinomycotic ones (Table 1). Actinomycotic grains are red or white elements with a diameter < 1 μm, thin and numerous, with fringes in the outskirt. The fungal ones are black measuring > 3 μm of diameter, with few thick hyphae dilated in places to form vesicles with no fringes, as seen in our patient [1] (Fig. 2). For the species diagnosis, the grains are seeded and deposited in tube culture media on Sabouraud-antibiotics culture media without actidione if they are fungal, or on Sabouraud without antibiotics or Loewenstein culture media if they are actinomycotic. Depending on the species, the cultures are more or less rapid to obtain. Species diagnosis is based on the macroscopic appearance of the cultures and microscopy (Fig. 3). In our patient, *Madurella mycetomatis* specific characteristics were identified on direct examination of grain and its culture. The grains are macroscopically black to brown firm grains from 0.5 to 1 mm. Direct microscopic examination is evocative and shows 3–4 μm septated hyphae branched in a network and ended in circular vesicles (chlamydoconidia) (Fig. 2)*. Madurella mycetomatis* culture grows slowly after at least 3 weeks at 27 °C on Sabouraud medium with antibiotics and without actidione. The culture macroscopic aspect is a circular flat felting colony with an elevated center and peripheral grey folds. The reverse side is dark to brown with a diffusible pigment in agar. The culture microscopic examination with Lactophenol Cotton Blue Stain shows septated blue hyphae and chlamydoconidia (Fig. 3). The absence of MALDI-TOF and PCR analysis are major limitations of the present report. The development of soft ionization techniques for mass spectrometry such as MALDI-TOF (*Matrix-Assisted Laser Desorption Ionization Time-Of-Flight*) allows a large panel analysis of specific species biomarkers. MALDI-TOF is more accurate than conventional phenotypic techniques in species identification with a lower cost per identification and a faster result. Indeed, MALDI-TOF is performed directly on samples without prior culture, which is useful for non-cultivable or slow-growing microorganisms [15]. Fungal DNA detection by PCR (*Polymerase Chain Reaction*) is also a technique used for species diagnosis. Molecular biology is performed on samples without a former culture. Unfortunately, it doesn’t always discriminate species, and other targeted genes are required. In addition to the high cost, this technique requires high expertise and should be reserved for non-cultivable microorganisms [16]. Moreover, MALDI-TOF and PCR were not used in our patient because of their lack of availability at the University Hospital of Rabat, Morocco. Their use would have decreased the required time for the species diagnosis but has no impact on the therapeutic choice in this case. Indeed, the black color of the grains on direct examination directs towards six possible fungal species (Table 2), and can already give a therapeutic orientation. It has been opted in our case for classical methods for species diagnosis (direct examination and grain culture) considering their low cost and accessibility, and their sensitivity and specificity. However, in endemic areas where diagnostic possibilities are limited, culture is rarely done and then the color and the macroscopic appearance of grains are used. The anatomopathological examination is particularly indicated when the patient is seen at a non-productive fistulas stage. Hematein-eosin staining is generally sufficient to study the grains, but depending on their appearance, specific staining may be required, and are helpful in differentiating organisms histopathologically: Periodic-acid–Schiff or Gomori-Grocott for fungi, Gram for actinomycetes. The reaction around the grains is generally granulomatous with granulocytes in contact with the grains, all surrounded by histiocytes and lymphocytes with neo-vessels. A third layer consists of fibrosis [17]. In our patient, the histopathology revealed the polymorphous inflammation. Moreover, medical imaging is particularly interesting in the assessment of mycetoma extension especially bone involvement, which is more frequent in actinomycetoma due to it being more osteophilic than fungal agents. Without treatment, this complication leads to functional impotence and can lead to amputation. Therefore standard radiography is compulsory and can show a cortical thinning or hypertrophy, bone cavities, and osteoporosis, which we didn’t find in our patient who had a fungal infection [18]. Ultrasonography is helpful for soft tissue extension. However, magnetic resonance imaging remains the gold standard to assess both the soft tissues and early bone involvements. Indeed, MRI shows the “dot-in-circle” sign (Fig. 4), corresponding to a central spherical hypointense signal (grains), surrounded by a hyperintense signal (the granuloma), with a peripheral low- signal matrix representing fibrous tissue [18]. Likewise, we found this sign in our patient as seen in Fig. 4, moreover, there was no bone involvement then no bone hypersignal.

The mycetoma treatment must be started as soon as possible before an advanced stage when amputation is the only therapeutic option. For that, there are much more therapeutic molecules to target actinomycetoma germs than eumycetoma ones. Moreover, eumycetoma is usually refractory to medications and an extended duration of treatment for at least 1 year is needed, against 3 months for bacterial mycetoma [19]. Indeed, we noticed in the first episode of mycetoma (ten years ago) in our patient a necessary duration of two years and a half for total healing of the fungal lesions. Moreover, for the current history, there is a slow improvement of his eumycetoma after 6 months of treatment. The molecules available for eumycetoma treatment include imidazoles such as ketoconazole (400 mg/day), itraconazole (200–400 mg/day), posaconazole (200 mg/day), voriconazole (400–600 mg/day); amphotericin B (0.5–1.25 mg/kg per day); and terbinafine (500–1000 mg/day), alone or in any combination [20]. For our patient, oral administration of terbinafine at a dosage of 750 mg/day was adopted because of its previous efficiency, availability and low cost comparing to other molecules. For actinomycetoma, trimethoprim-sulfamethoxazole is the most effective antibiotic [21]. On the other hand, surgical tumorectomy is performed in small, localized lesions, and also for large lesions to reduce the mass size for better medical treatment effectiveness [22]. Indeed, relapses can occur and a follow-up for many years is needed to ensure complete remission of the disease. Our case report is a good illustration of the possible late relapses, seen here after ten years of complete remission. Prevention remains the best treatment and is based on simple measures such as wearing protective garments and shoes, especially in rural areas and during outdoor activities.

In conclusion, mycetoma is a chronic granulomatous infection of cutaneous and subcutaneous tissues that can be caused by filamentous bacteria and less frequently fungi. It is a rare neglected tropical disease, and Morocco is a non-endemic country. Therefore the diagnosis of “Madura foot” isn’t in the foreground in patients presenting with a polyfistulised mass of the foot. Cutaneous tuberculosis remains the first evocated diagnosis leading to delay presentation were amputation is the only therapeutic option. This case report is in accordance with the literature data, and it should raise awareness of this uncommon disease that is now appearing in non-endemic countries.

## Data Availability

Data sharing is not applicable to this article as no datasets were generated or analyzed during the current study.

## Abbreviations

MALDI-TOF: Matrix-Assisted Laser Desorption Ionization Time-Of-Flight
MRI: Magnetic Resonance Imaging
PCR: Polymerase Chain Reaction
